# Editorial: Environmental or occupational exposure to optical radiation: Risk evaluation, health effects and prevention - tangible innovation for public and occupational health?

**DOI:** 10.3389/fpubh.2022.969245

**Published:** 2022-07-18

**Authors:** Alberto Modenese

**Affiliations:** Department of Biomedical, Metabolic and Neural Sciences, University of Modena and Reggio Emilia, Modena, Italy

**Keywords:** ultraviolet radiation, skin cancer, cataract, dosimetry, occupational health, occupational exposure, environmental exposure, heat

This Special Issue addresses the Research Topic of exposure to optical radiation (OR), considering in particular solar radiation and the health consequences of an excessive exposure, the issues related to risk evaluation and the indications for an appropriate prevention of this environmental and occupational hazard. The Sun emits all the types of OR, including infrared, visible and ultraviolet radiation (UVR) (1). This latter is the most harmful component of OR, able to induce not only short-term adverse effects mainly at the eyes and the skin, but also long term ones, including cancers (2). With regard to outdoor workers (OW) exposed to solar UVR, scientific literature proves a high burden of cancers related to this exposure, especially keratinocytes carcinomas, as recently reported in a systematic review (3), even if these pathologies are often under-recognized, when not totally neglected, as “occupational diseases” (2, 3). From an occupational hazard perspective, UVR is currently acknowledged as the occupational carcinogenic agent the most subjects are exposed to, and it is also a known risk factors for various other eye and skin acute and chronic diseases: the acute ones include sunburns, photoconjunctivitis and photokeratitis, while among the chronic ones there are the above-mentioned skin cancers, as well as pterygium and cataract for the eyes. These diseases are included in the overview on health risks associated with excessive exposure to solar UVR among OW by Wright and Norval, published under this Special Issue and with a specific focus on a Country as South Africa, for which up to now only a few reports on work-related OR risk were available. Moreover, in most Countries of the world there are no recognized criteria for the recognition and prevention of UVR-related occupational skin cancers, as well as no valid exposure limit values for solar UVR exposure, as highlighted by Wittlich, who proposes here a series of brand new criteria of occupational health prevention for solar UVR exposed OW. Wittlich also underlines that it is extremely important to conduct extensive and rigorous measurements campaigns to identify solar UVR exposure levels posing OW at risk for adverse effects. This was done by Heepenstrick et al., who showed different reliable approaches for an effective dosimetry to be applied in various outdoor activities. With regard to the exposure of the eyes, Marro et al. reviewed the available methods reported in literature, along with their limitations, to study ocular UVR exposure and its implications for health.

Shifting to prevention of adverse effects, one of the main topics currently addressed in scientific research is the studying of the most effective interventions to be applied for the protection of OW and of the general public, with the final aim of reducing the burden of skin cancers. This is the topic of a systematic review being currently conducted by an international research group, with its protocol registered in PROSPERO and fully published under the present Special Issue (Modenese et al.). In addition, a specific intervention for Dutch construction workers has been designed by Keurentjes et al.(a), who first published the protocol for their non-randomized intervention study, and then reported here the first results collected with a pilot study aimed at stimulating the use of sunscreen among construction workers. This study reveals that, even if provided, construction workers scarcely use sunscreen, although they report of being sufficiently informed on solar UVR risk and those using sunscreens seem satisfied with them by Keurentjes et al.(b),. The results of another European intervention in this field are reported in the Danish study by Jacobsen et al., indicating that the awareness of occupational skin cancer risk and the perception of the importance of prevention and sun protection at work amongst outdoor workers can be improved with a specific multicomponent intervention.

Finally, the present Special Issue also reminds us that we are in the climate changing era, and Sun-related occupational and environmental hazards are not limited to solar UVR: as a matter of fact, heat waves and their possible adverse health consequences are becoming a serious concern for the performance of occupational and leisure outdoor activities worldwide: in this context, the study conducted by Wang et al. highlights an insufficient awareness of military personnel in China with respect to preventive and first-aid measures against heat-related illnesses, indicating, as it happens also for UVR-related effects, an urgent need of targeted educational interventions.

## Author contributions

The author confirms being the sole contributor of this work and has approved it for publication.

## Conflict of interest

The author declares that the research was conducted in the absence of any commercial or financial relationships that could be construed as a potential conflict of interest.

## Publisher's note

All claims expressed in this article are solely those of the authors and do not necessarily represent those of their affiliated organizations, or those of the publisher, the editors and the reviewers. Any product that may be evaluated in this article, or claim that may be made by its manufacturer, is not guaranteed or endorsed by the publisher.
